# Quality of ultrasound biometry obtained by local health workers in a refugee camp on the Thai–Burmese border

**DOI:** 10.1002/uog.11091

**Published:** 2012-07-30

**Authors:** M J Rijken, E J H Mulder, A T Papageorghiou, S Thiptharakun, N Wah, T K Paw, S L M Dwell, G H A Visser, F H Nosten, R McGready

**Affiliations:** * Shoklo Malaria Research UnitMae Sot, Tak, Thailand; † Department of Perinatology & Gynecology, University Medical Center UtrechtUtrecht, The Netherlands; ‡ Nuffield Department of Obstetrics & Gynaecology, University of OxfordOxford, UK; § Mahidol-Oxford Tropical Medicine Research Unit (MORU), Mahidol UniversityBangkok, Thailand; ¶ Centre for Tropical Medicine, University of OxfordOxford, UK

**Keywords:** developing country, fetal biometry, health workers, quality, reference equations, standard deviation, Thai–Burmese border, ultrasound, *Z*-score

## Abstract

**Objective:**

In a refugee camp on the Thai–Burmese border, accurate dating of pregnancy relies on ultrasound measurements obtained by locally trained health workers. The aim of this study was to substantiate the accuracy of fetal biometry measurements performed by locally trained health workers by comparing derived reference equations with those published for Asian and European hospitals.

**Methods:**

This prospective observational study included 1090 women who had a dating crown–rump length (CRL) scan and one study-appointed ultrasound biometry scan between 16 and 40 weeks of gestation. The average of two measurements of each of biparietal diameter, head circumference, abdominal circumference and femur length was used in a polynomial regression model for the mean and SD against gestational age (GA). The biometry equations obtained were compared with published equations of professional sonographers from Asian and European hospitals by evaluation of the SD and *Z*-scores of differences between models.

**Results:**

Reference equations of biometric parameters were found to fit cubic polynomial models. The observed SD values, for any given GA, of fetal biometric measurements obtained by locally trained health workers were lower than those previously reported by centers with professional sonographers. For nearly the entire GA range considered, the mean values of the Asian and European equations for all four biometric measurements were within the 90% expected range (mean ± 1.645 SD) of our equations.

**Conclusion:**

Locally trained health workers in a refugee camp on the Thai–Burmese border can obtain measurements that are associated with low SD values and within the normal limits of published Asian and European equations. The fact that the SD values were lower than in other studies may be explained by the use of the average of two measurements, CRL dating or motivation of the locally trained sonographers. Copyright © 2012 ISUOG. Published by John Wiley & Sons, Ltd.

## Introduction

Assessment of fetal growth is an important component of antenatal care, and precise dating is crucial for detection of subsequent growth restriction. Although accurate gestational age (GA) assessment is not a problem unique to resource-poor settings^1^–^3^, a large proportion of women in such settings are unable to give reliable last menstrual period (LMP) dates^4^. For example, in the antenatal clinics on the Thai–Burmese border, less than a third of women are able to confirm their LMP^5^. Explanations for this include: GA is counted in months, and attempts to translate this to a LMP date are complicated and usually inaccurate; literacy levels in pregnant women in Maela Refugee camp are less than 50%^6^; and there are several calendars in use (standard western, Thai, Karen, Burmese and Buddhist)^5^. Accordingly, dating of pregnancy relies on ultrasound measurements, made ideally in the first trimester.

Local health workers are trained as sonographers. We have shown that these staff, with limited or no tertiary education, can achieve high levels of accuracy in GA assessment after a 3-month training course, including on-the-job training and ongoing quality control (QC) measures^5^. This ongoing QC system is in place for dating scans in the first-trimester (using crown–rump length (CRL)) and second-trimester biometry scans. The aim of this study was to substantiate the accuracy of the health workers' fetal biometry measurements by comparing the *Z*-scores and associated SD of biometry equations created for this purpose with those from published equations of professional sonographers from Asian and European hospitals.

## Methods

### Study site and population

The Shoklo Malaria Research Unit (SMRU) is located on the Thai–Burmese border and has five established clinics, one of which is based in Maela refugee camp. The main population in this camp belongs to the Karen ethnic group. Details of the SMRU antenatal clinics are described in full elsewhere^5^, ^7^. In short, the antenatal clinics were commenced in 1986 and ultrasound scanning was introduced in 2001. All pregnant women are encouraged to attend the antenatal clinic as early as possible in the first trimester of pregnancy. At the first visit, ultrasound imaging is used to determine viability, detect multiple pregnancy and estimate GA. Routinely, a second scan is performed at 18–24 weeks to reassess viability, measure fetal biometry, identify major fetal abnormalities and determine placental position.

### Study procedures

Women who attended the SMRU antenatal clinic in Maela refugee camp were invited to consent to have an additional biometry scan performed at a specific GA in order to assess image quality at any time in pregnancy as part of the ongoing QC of local sonographers^5^. The data for this study were collected from this QC program of the same group of sonographers, with four participating at any given time. Two sonographers left the refugee camp in 2009 for resettlement into a western country and were replaced with two newly trained sonographers. One of the sonographers had completed 3 years of training as a nurse at a recognized institution in Burma. The others did not have any tertiary education but had completed school to grade 10 (16 years old). All sonographers had at least 12 months of work experience before participating in this data collection and, during each month in 2010, together they scanned a median of 439 (range, 340–492) women. The focus on image and measurement quality for sonographers was part of the preparation and training for a fetal growth study (ClinicalTrials.gov Identifier: NCT00840502), approved by Oxford University (OxTREC (14-08)) and Mahidol University (TMEC 2008-028) Ethics Committees.

Women with a live singleton fetus who had an early dating ultrasound (defined as a CRL measurement of 8–79 mm, corresponding to 7–14 weeks of gestation) were assigned to return for a study scan between 16 and 40 weeks, at which biparietal diameter (BPD), head circumference (HC), abdominal circumference (AC) and femur length (FL) were measured. This study scan was in addition to their routine second-trimester scan. When a woman did not attend for the appointed study scan, the data of the routine biometry measurements were used. Pregnancies complicated by serious infectious diseases (e.g. malaria) before the scan, and pregnancies that had an unknown outcome or resulted in stillbirth, were excluded. No fetuses were excluded on the basis of abnormal biometry, birth weight, preterm delivery or congenital abnormality (of which there were six cases: one with sacrococcygeal teratoma; two with skin tags; one with Down syndrome; one with syndactyly; and one with cleft palate).

The training manual and protocol for obtaining transabdominal CRL and biometry measurements were according to the British Medical Ultrasound Society recommendations^8^; the BPD is measured locally in the plane of the HC by placing the calipers on the outer border of the upper and the inner borders of the lower parietal bones (‘outer to inner’, BPD) across the widest part of the skull. All scans were performed with no time constraints, in a room on a reinforced bamboo floor, by four locally trained sonographers^5^ using a Toshiba Powervision 7000 machine (Toshiba, Tokyo, Japan) with a 3.75-MHz convex probe, which was donated by the University of Utrecht, The Netherlands. Owing to electrical surges in the refugee camp, a voltage stabilizer was used to operate the ultrasound scanner. At each scan, the measurements, recorded in mm, were obtained twice and the examiners were blinded to the expected GA and the results of the examinations.

### Statistical analysis

Data were entered into a Microsoft Access database and statistical analysis was performed using SPSS version 15.0 for Windows (SPSS Inc., Chicago, IL, USA) and Microsoft Excel. The mean of the two first-trimester CRL measurements was used to define the GA^9^. Each woman provided just one designated biometry examination, and the mean of two measurements for each biometric parameter was included for analysis. In order to obtain reference ranges for fetal measurements, a polynomial regression model was used, as recommended previously^10^, ^11^. Least-squares regression analysis was used to model the mean by fitting a polynomial equation, including a linear, quadratic and cubic component for all measurements. The variability in measurements was modeled by computing the SD at each week of gestation, and the SD values were regressed on GA using a linear equation. From the predictive mean and SD, equation centiles were calculated using the formula:


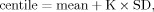


where K is the corresponding centile of the standard normal distribution: ± 1.88 for 3^rd^ and 97^th^ centiles, and ± 1.28 for 10^th^ and 90^th^ centiles. Charts were computed by plotting predicted means and 3^rd^, 10^th^, 50^th^, 90^th^ and 97^th^ centiles against GA.

For each GA between 16 and 40 weeks, the biometric measurements of this study were compared with published equations from Asian^12^, ^13^ and European^14^–^18^ hospitals using the *Z*-score method^19^–^21^. The data were expressed as *Z*-scores using the formula:


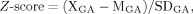


where X_GA_ is the mean value from other populations at a known GA, M_GA_ is the mean value for the study population calculated from our equation at this GA and SD_GA_ is the SD associated with the mean value at the same GA from our population. The *Z*-scores and the published SD of each equation are presented graphically across the different GAs to allow visual comparison.

## Results

Between April 2007 and October 2010, 1090 women with a live singleton newborn were included in this prospective observational study. Median (interquartile range) age, gravidity and mid upper-arm circumference (MUAC) were 25 (20–30) years, one (1–4) and 24.0 (23.0–26.0) cm, respectively. The mean (SD) birth weight in this group was 3017 (428) g and mean GA at delivery was 39.1 (1.6) weeks. The median number of examinations performed at each week of gestation was 39 (interquartile range, 32–45) (Table S1).

The raw data were fitted satisfactorily with a cubic polynomial model for all biometric parameters, as follows (all measurements in mm and GA in exact weeks):


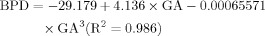



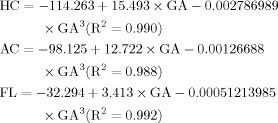


Data analysis showed that the addition of a quadratic component did not improve the fit of the curves. The corresponding equations for the SD fitted in a linear equation:


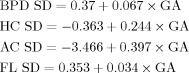


**Figure 1:**
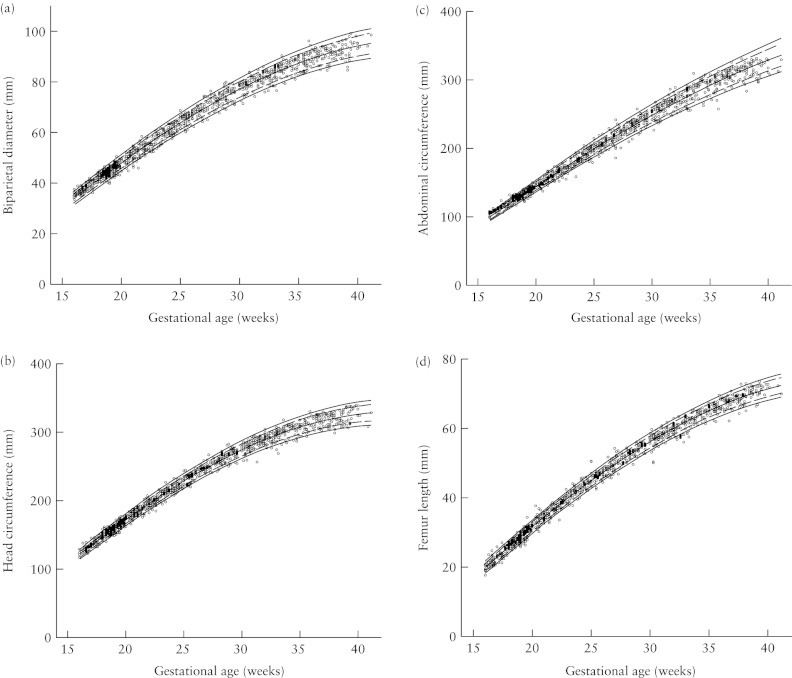
Raw data (*n* = 1090) with 3^rd^, 10^th^, 50^th^, 90^th^ and 97^th^ fitted centiles for biparietal diameter (a), head circumference (b), abdominal circumference (c) and femur length (d).

The goodness-of-fit of the model was assessed using raw data for each measurement (Figure 1). Figures S1–S4 and Tables S2–S5 present the charts and tables for clinical use with the 3^rd^, 10^th^, 50^th^, 90^th^ and 97^th^ percentiles of BPD, HC, AC and FL.

**Figure 2:**
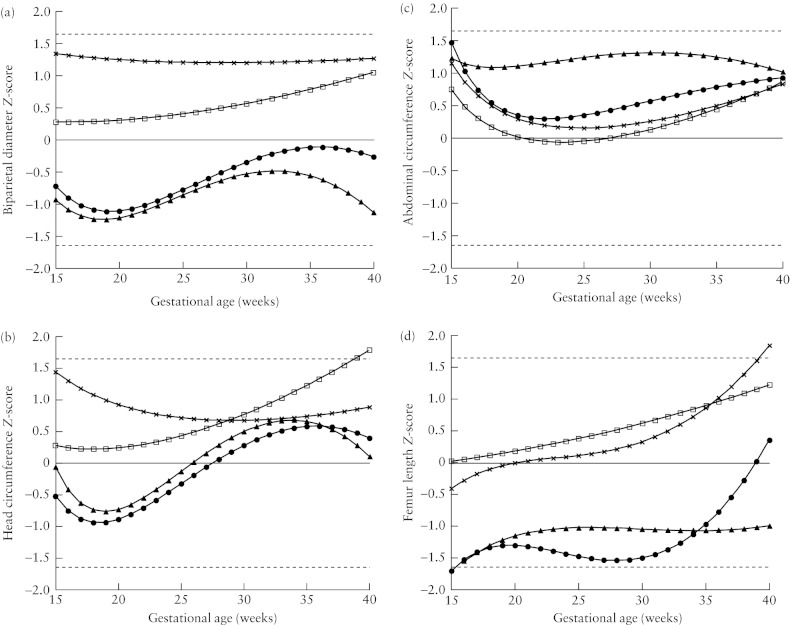
*Z*-score comparison of the equations of the present study with Asian (China^13^ (•), Korea^12^ (▴)) and European (Switzerland^14^, ^15^ (×) and the UK^16^–^18^ (□)) equations for mean biparietal diameter (a), head circumference (b), abdominal circumference (c) and femur length (d). Mean expected *Z*-score or 50^th^ percentile is shown as solid line; dashed lines represent the expected *Z*-scores for the 5^th^ and 95^th^ centiles (i.e. − 1.645 and 1.645, respectively).

Figure 2 shows the 50^th^ centiles of each biometric parameter from previously published equations, expressed as *Z*-scores based on our equations^12^–^18^. If the measurements from all populations were identical, Figure 2 would show one line at *y* = 0. For nearly the entire GA range the mean values of the Asian and European equations for all four biometric measurements were between the 5^th^ (mean − 1.645 SD) and 95^th^ (mean + 1.645 SD) centiles of our equations. The mean AC of fetuses in this study was smaller throughout the pregnancy than that of any of the other equations, and the mean HC was smaller in the second half of pregnancy.

**Figure 3:**
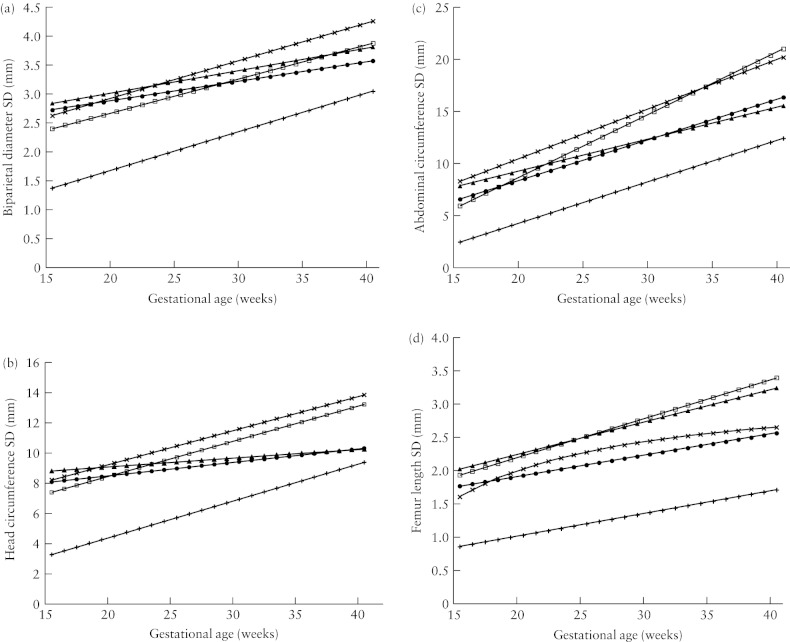
Comparison of the SD equations of the present study (+) with Asian (China^13^ (•), Korea^12^ (▴)) and European (Switzerland^14^, ^15^ (×) and the UK^16^–^18^ (□)) SD equations of biparietal diameter (a), head circumference (b), abdominal circumference (c) and femur length (d).

When comparing the SD for this population with the SD generated from the equations of Asian^12^, ^13^ and European^14^–^18^ studies, it can be observed that, for any given GA, the SD was significantly smaller in this study population (Figure 3).

## Discussion

Antenatal ultrasound is increasingly seen as a useful adjunct to obstetric care in resource-poor settings: ultrasound machines are becoming more readily available^22^–^24^ and locally trained health workers can obtain accurate fetal biometry measurements for GA estimation^5^, ^25^. Accurate pregnancy dating is one of the obvious benefits in populations where the LMP is not available or cannot reliably be obtained. However, the value of antenatal ultrasound depends on appropriate use by adequately trained sonographers with ongoing QC support in settings with the necessary infrastructure^5^, ^24^. This study confirms that very motivated locally trained health workers can successfully obtain biometry measurements between 16 and 40 weeks of GA. The low SD values derived from the reference equations suggest that the quality of the measurements is associated with a low random error.

The choice of reference charts and equations for fetal size has an impact on the quality of fetal biometry in clinical practice^11^, ^19^. There have been concerns about incorrect methods that are being used to estimate age-specific reference intervals (‘normal ranges’) for fetal measurements^11^. Country-specific differences in, for example, caliper placement, have been highlighted to explain differences between reference charts and equations within European studies^20^. Midwives had a greater tendency than physicians to normalize biometry data^26^. In this study, the examiners were blinded to the estimated GA and their measurement, so such a normalization of data is unlikely to have occurred. Furthermore, all sonographers received the same training and followed the strict guidelines for ultrasound examination, including the plane of the measurement and caliper placement.

When *Z*-scores were used to compare the reference equations with previously published equations from two Asian (China^13^ and Korea^12^) and two European (Switzerland^14^, ^15^ and UK^16^–^18^) studies that also provided BPD outer-to-inner measurements, several points can be made (Figure 2). The fact that the *Z*-scores of the other equations were within the 5% and 95% range of our equation suggests that the locally trained sonographers are able to obtain measurements that are comparable with those of expert sonographers. Generally, the *Z*-scores of Asian fetuses appear smaller than the *Z*-scores obtained in European studies. The smaller AC of Karen fetuses throughout pregnancy compared with those of the other populations may reflect the socio-economic conditions in the refugee camp. In this study, the FL was smaller compared with the FL of European fetuses, which is in agreement with previously published articles, where it was explained by racial differences^12^, ^13^.

From Figure 3, the most striking difference is the smaller SD at any GA for this study's equations. By definition, SD shows the variation or ‘dispersion’ from the mean (or expected value) and depends on measurement error as well as true variation between subjects. One explanation could be that in this study the equations were based on the mean of two measurements for both the CRL and the biometry measurements, which reduces variation from the expected value. Also in this study, pregnancy dating was based on first-trimester CRL, which results in less variation of GA than when LMP is used for pregnancy dating^2^, ^27^. In the four other studies, GA was only corrected to the ultrasound value if the difference between the GA estimated by CRL and the GA estimated by LMP exceeded 4^13^, 5^15^ or 10^12^, ^18^ days. On the other hand, this may also be seen as a limitation of our study because the use of a CRL measurement at a single time-point for dating does not account for first-trimester growth restriction^28^–^30^. Nevertheless, in the absence of reliable LMP dates in our population, dating by CRL is the most appropriate method. The SD, being the denominator of the formula, has an important impact on the magnitude of a *Z*-score. When the *Z*-scores were calculated based on the SD of another equation^16^–^18^, the *Z*-scores were all closer to zero (see Figure S5). To put *Z*-scores into clinical context, a mathematical example was created. For an examiner measuring a fetus at exactly 20 weeks' GA, the expected mean HC, SD and 95% prediction interval in mm of the five equations are shown in Table 1.

**Table 1:** Expected head circumference measurement at 20 + 0 weeks for each of the different equations

	Expected head circumference (mm)	
	
Equation^reference^ (study sample size)	Mean ± SD	95% PI
Present study (*n* = 1090)	173.3 ± 4.5	164.5–182.1
Switzerland^15^ (*n* = 6557)	177.5 ± 9.4	159.1–195.9
UK^18^ (*n* = 663)	174.4 ± 8.5	157.7–191.1
China^13^ (*n* = 709)	169.3 ± 8.5	152.6–186.0
Korea^12^ (*n* = 10 455)	170.0 ± 9.1	152.2–187.8

PI, prediction interval.

In conclusion, SD and *Z*-score comparison can be used to assess QC, and this study suggests that locally trained health workers can obtain measurements that are associated with a low SD and are within the normal limit of Asian and European equations. The fact that the SD values were even lower than those of other studies may have various explanations, including that the average of two measurements was used for both CRL and biometry, that CRL dating, and not the LMP method, was used for pregnancy dating and that locally trained sonographers were particularly motivated. Further research is needed to assess the relative impact of these possible factors in other populations.

## SUPPORTING INFORMATION ON THE INTERNET

The following supporting information may be found in the online version of this article:

**Figures S1–S4** Fetal biometric charts with 3^rd^, 10^th^, 50^th^, 90^th^ and 97^th^ fitted centiles in a Karen population in a refugee camp on the Thai–Burmese Border: biparietal diameter (Figure S1), head circumference (Figure S2), abdominal circumference (Figure S3) and femur length (Figure S4).

**Figure S5** *Z*-scores for comparison of fetal biometry with Asian and European equations.

**Table S1** Number of observations according to gestational age in completed weeks in singleton pregnancies on the Thai–Burmese border.

**Tables S2–S5** Fitted biometry centiles in a Karen population in a refugee camp on the Thai–Burmese border: biparietal diameter (Table S2), head circumference (Table S3), abdominal circumference (Table S4) and femur length (Table S5).
