# Supplementary material for: Quality of ultrasound biometry obtained by local health workers in a refugee camp on the Thai–Burmese border
Source: Ultrasound Obstet Gynecol. 2012 Jul 30;40(2):151–7. doi: 10.1002/uog.11091 (PMC3443371; doi:10.1002/uog.11091)
Supplement: Supplementary file 1 [file uog0040-0151-SD1.doc]

Supplementary figure S1, S2, S3, S4

Fetal biometric charts with 3^rd^, 10^th^, 50^th^, 90^th^, and 97^th^ fitted centiles in a Karen population in a refugee camp on the Thai-Burmese Border:

S1 biparietal diameter (outer–inner; BPD)

S2 head circumference (HC)

S3 abdominal circumference (AC)

S4 femur length (FL)

Supplementary figure S1

Supplementary figure S2

Supplementary figure S3

Supplementary figure S4
