# Supplementary material for: Quality of ultrasound biometry obtained by local health workers in a refugee camp on the Thai–Burmese border
Source: Ultrasound Obstet Gynecol. 2012 Jul 30;40(2):151–7. doi: 10.1002/uog.11091 (PMC3443371; doi:10.1002/uog.11091)
Supplement: Supplementary file 2 [file uog0040-0151-SD2.doc]

Supplementary figure S5

Z-scores for comparison of fetal biometry with Asian and European equations

Comparison of the Switzerland^1, 2^ (×), China^3^ (●), Korea^4^ (▲) and present study (+) equations with the UK^5-7^ equations for mean BPD, HC, AC and FL.

Mean expected z-score, or 50^th^ percentile is shown as straight black line, dashes lines represent the expected z-scores for the 5^th^ and 95^th^ centiles, i.e. -1.645 and 1.645 respectively. The x-axis shows the gestational age in weeks, the y-axis the z-score.

References

1. J. Kurmanavicius, E. M. Wright, P. Royston, R. Zimmermann, R. Huch, A. Huch and J. Wisser. Fetal ultrasound biometry: 2. Abdomen and femur length reference values. *Br J Obstet Gynaecol* 1999; **106**: 136-143.

2. J. Kurmanavicius, E. M. Wright, P. Royston, J. Wisser, R. Huch, A. Huch and R. Zimmermann. Fetal ultrasound biometry: 1. Head reference values. *Br J Obstet Gynaecol* 1999; **106**: 126-135.

3. T. N. Leung, M. W. Pang, S. S. Daljit, T. Y. Leung, C. F. Poon, S. M. Wong and T. K. Lau. Fetal biometry in ethnic Chinese: biparietal diameter, head circumference, abdominal circumference and femur length. *Ultrasound Obstet Gynecol* 2008; **31**: 321-327.

4. S. I. Jung, Y. H. Lee, M. H. Moon, M. J. Song, J. Y. Min, J. A. Kim, J. H. Park, J. H. Yang, M. Y. Kim, J. H. Chung, J. Y. Cho and K. G. Kim. Reference charts and equations of Korean fetal biometry. *Prenat Diagn* 2007; **27**: 545-551.

5. L. S. Chitty, D. G. Altman, A. Henderson and S. Campbell. Charts of fetal size: 4. Femur length. *Br J Obstet Gynaecol* 1994; **101**: 132-135.

6. L. S. Chitty, D. G. Altman, A. Henderson and S. Campbell. Charts of fetal size: 3. Abdominal measurements. *Br J Obstet Gynaecol* 1994; **101**: 125-131.

7. L. S. Chitty, D. G. Altman, A. Henderson and S. Campbell. Charts of fetal size: 2. Head measurements. *Br J Obstet Gynaecol* 1994; **101**: 35-43.
