# Supplementary material for: Quality of ultrasound biometry obtained by local health workers in a refugee camp on the Thai–Burmese border
Source: Ultrasound Obstet Gynecol. 2012 Jul 30;40(2):151–7. doi: 10.1002/uog.11091 (PMC3443371; doi:10.1002/uog.11091)
Supplement: Supplementary file 3 [file uog0040-0151-SD3.doc]

Supplementary Table S1

Number of observations according to gestational age (GA) in completed weeks in singleton pregnancies on the Thai Burmese border

| GA | Number of women scanned |
| --- | --- |
| 16 | 39 |
| 17 | 37 |
| 18 | 134 |
| 19 | 123 |
| 20 | 41 |
| 21 | 50 |
| 22 | 47 |
| 23 | 42 |
| 24 | 45 |
| 25 | 45 |
| 26 | 42 |
| 27 | 32 |
| 28 | 41 |
| 29 | 30 |
| 30 | 46 |
| 31 | 38 |
| 32 | 39 |
| 33 | 39 |
| 34 | 30 |
| 35 | 33 |
| 36 | 38 |
| 37 | 30 |
| 38 | 26 |
| 39 | 17 |
| 40 | 6 |
