# Supplementary material for: Quality of ultrasound biometry obtained by local health workers in a refugee camp on the Thai–Burmese border
Source: Ultrasound Obstet Gynecol. 2012 Jul 30;40(2):151–7. doi: 10.1002/uog.11091 (PMC3443371; doi:10.1002/uog.11091)
Supplement: Supplementary file 4 [file uog0040-0151-SD4.doc]

Supplementary tables S2, S3, S4, S5

Fitted biometry centiles in a Karen population in a refugee camp on the Thai-Burmese Border:

S2 biparietal diameter (outer–inner; BPD)

S3 head circumference (HC)

S4 abdominal circumference (AC)

S5 femur length (FL)

Table S2

Biparietal diameter (outer to inner) in mm

| **Weeks of gestation** | **Percentiles** | | | | | | | | | | |
| --- | --- | --- | --- | --- | --- | --- | --- | --- | --- | --- | --- |
|  | **P2.3** | **P3** | **P5** | **P10** | **P25** | **P50** | **P75** | **P90** | **P95** | **P97** | **P97.7** |
| **15** | 28.0 | 28.1 | 28.4 | 28.9 | 29.7 | 30.6 | 31.6 | 32.4 | 32.9 | 33.2 | 33.3 |
| **16** | 31.5 | 31.6 | 31.9 | 32.5 | 33.3 | 34.3 | 35.3 | 36.2 | 36.7 | 37.0 | 37.1 |
| **17** | 35.0 | 35.1 | 35.4 | 36.0 | 36.9 | 37.9 | 38.9 | 39.8 | 40.4 | 40.7 | 40.9 |
| **18** | 38.4 | 38.5 | 38.8 | 39.4 | 40.4 | 41.4 | 42.5 | 43.5 | 44.0 | 44.4 | 44.5 |
| **19** | 41.7 | 41.8 | 42.2 | 42.8 | 43.8 | 44.9 | 46.0 | 47.0 | 47.6 | 48.0 | 48.1 |
| **20** | 44.9 | 45.1 | 45.5 | 46.1 | 47.1 | 48.3 | 49.4 | 50.5 | 51.1 | 51.5 | 51.6 |
| **21** | 48.1 | 48.3 | 48.7 | 49.3 | 50.4 | 51.6 | 52.8 | 53.9 | 54.5 | 54.9 | 55.1 |
| **22** | 51.2 | 51.4 | 51.8 | 52.5 | 53.6 | 54.8 | 56.1 | 57.2 | 57.9 | 58.3 | 58.4 |
| **23** | 54.2 | 54.4 | 54.8 | 55.5 | 56.7 | 58.0 | 59.3 | 60.4 | 61.1 | 61.6 | 61.7 |
| **24** | 57.1 | 57.3 | 57.8 | 58.5 | 59.7 | 61.0 | 62.3 | 63.6 | 64.3 | 64.7 | 64.9 |
| **25** | 60.0 | 60.1 | 60.6 | 61.4 | 62.6 | 64.0 | 65.3 | 66.6 | 67.3 | 67.8 | 68.0 |
| **26** | 62.7 | 62.9 | 63.3 | 64.1 | 65.4 | 66.8 | 68.2 | 69.5 | 70.3 | 70.8 | 71.0 |
| **27** | 65.3 | 65.5 | 66.0 | 66.8 | 68.1 | 69.6 | 71.0 | 72.4 | 73.2 | 73.7 | 73.9 |
| **28** | 67.8 | 68.0 | 68.5 | 69.4 | 70.7 | 72.2 | 73.7 | 75.1 | 75.9 | 76.5 | 76.6 |
| **29** | 70.2 | 70.4 | 71.0 | 71.8 | 73.2 | 74.8 | 76.3 | 77.7 | 78.6 | 79.1 | 79.3 |
| **30** | 72.5 | 72.7 | 73.3 | 74.2 | 75.6 | 77.2 | 78.8 | 80.2 | 81.1 | 81.7 | 81.9 |
| **31** | 74.7 | 74.9 | 75.5 | 76.4 | 77.9 | 79.5 | 81.1 | 82.6 | 83.5 | 84.1 | 84.3 |
| **32** | 76.8 | 77.0 | 77.5 | 78.5 | 80.0 | 81.7 | 83.4 | 84.9 | 85.8 | 86.4 | 86.6 |
| **33** | 78.7 | 78.9 | 79.5 | 80.4 | 82.0 | 83.7 | 85.5 | 87.0 | 88.0 | 88.6 | 88.8 |
| **34** | 80.5 | 80.7 | 81.3 | 82.3 | 83.9 | 85.7 | 87.4 | 89.1 | 90.0 | 90.7 | 90.9 |
| **35** | 82.1 | 82.4 | 83.0 | 84.0 | 85.6 | 87.5 | 89.3 | 90.9 | 91.9 | 92.6 | 92.8 |
| **36** | 83.7 | 83.9 | 84.5 | 85.6 | 87.3 | 89.1 | 91.0 | 92.7 | 93.7 | 94.4 | 94.6 |
| **37** | 85.1 | 85.3 | 85.9 | 87.0 | 88.7 | 90.6 | 92.5 | 94.3 | 95.3 | 96.0 | 96.2 |
| **38** | 86.3 | 86.5 | 87.2 | 88.3 | 90.1 | 92.0 | 94.0 | 95.7 | 96.8 | 97.5 | 97.7 |
| **39** | 87.4 | 87.6 | 88.3 | 89.4 | 91.2 | 93.2 | 95.2 | 97.0 | 98.2 | 98.8 | 99.1 |
| **40** | 88.3 | 88.6 | 89.3 | 90.4 | 92.3 | 94.3 | 96.3 | 98.2 | 99.3 | 100.0 | 100.3 |
|  |  |  |  |  |  |  |  |  |  |  |  |

Table S3

Head circumference in mm

| **Weeks of gestation** | **Percentiles** | | | | | | | | | | |
| --- | --- | --- | --- | --- | --- | --- | --- | --- | --- | --- | --- |
|  | **P2.3** | **P3** | **P5** | **P10** | **P25** | **P50** | **P75** | **P90** | **P95** | **P97** | **P97.7** |
| **15** | 102.3 | 102.5 | 103.3 | 104.5 | 106.5 | 108.7 | 110.9 | 112.9 | 114.2 | 114.9 | 115.2 |
| **16** | 115.3 | 115.6 | 116.4 | 117.7 | 119.8 | 122.2 | 124.6 | 126.7 | 128.1 | 128.9 | 129.1 |
| **17** | 128.0 | 128.3 | 129.2 | 130.6 | 132.9 | 135.4 | 138.0 | 140.3 | 141.7 | 142.5 | 142.8 |
| **18** | 140.5 | 140.8 | 141.7 | 143.2 | 145.7 | 148.4 | 151.1 | 153.5 | 155.0 | 155.9 | 156.3 |
| **19** | 152.6 | 153.0 | 153.9 | 155.5 | 158.1 | 161.0 | 163.9 | 166.5 | 168.0 | 169.0 | 169.4 |
| **20** | 164.4 | 164.8 | 165.8 | 167.5 | 170.3 | 173.3 | 176.3 | 179.1 | 180.8 | 181.8 | 182.2 |
| **21** | 175.9 | 176.3 | 177.4 | 179.2 | 182.1 | 185.3 | 188.5 | 191.4 | 193.1 | 194.2 | 194.6 |
| **22** | 187.1 | 187.5 | 188.6 | 190.5 | 193.6 | 196.9 | 200.3 | 203.3 | 205.2 | 206.3 | 206.7 |
| **23** | 197.9 | 198.3 | 199.5 | 201.4 | 204.6 | 208.2 | 211.7 | 214.9 | 216.8 | 218.0 | 218.5 |
| **24** | 208.3 | 208.7 | 210.0 | 212.0 | 215.4 | 219.0 | 222.7 | 226.1 | 228.1 | 229.4 | 229.8 |
| **25** | 218.3 | 218.7 | 220.0 | 222.2 | 225.7 | 229.5 | 233.4 | 236.9 | 239.0 | 240.3 | 240.8 |
| **26** | 227.8 | 228.3 | 229.7 | 231.9 | 235.6 | 239.6 | 243.6 | 247.2 | 249.4 | 250.8 | 251.3 |
| **27** | 237.0 | 237.5 | 238.9 | 241.2 | 245.0 | 249.2 | 253.4 | 257.2 | 259.5 | 260.9 | 261.4 |
| **28** | 245.7 | 246.2 | 247.7 | 250.1 | 254.0 | 258.4 | 262.7 | 266.6 | 269.0 | 270.5 | 271.0 |
| **29** | 253.9 | 254.4 | 256.0 | 258.5 | 262.6 | 267.1 | 271.6 | 275.7 | 278.1 | 279.7 | 280.2 |
| **30** | 261.6 | 262.2 | 263.8 | 266.4 | 270.6 | 275.3 | 279.9 | 284.2 | 286.8 | 288.4 | 288.9 |
| **31** | 268.9 | 269.5 | 271.1 | 273.8 | 278.2 | 283.0 | 287.8 | 292.2 | 294.9 | 296.5 | 297.1 |
| **32** | 275.6 | 276.2 | 277.9 | 280.7 | 285.2 | 290.2 | 295.2 | 299.7 | 302.5 | 304.2 | 304.8 |
| **33** | 281.8 | 282.4 | 284.2 | 287.0 | 291.7 | 296.8 | 302.0 | 306.7 | 309.5 | 311.3 | 311.9 |
| **34** | 287.4 | 288.0 | 289.9 | 292.8 | 297.6 | 303.0 | 308.3 | 313.1 | 316.0 | 317.9 | 318.5 |
| **35** | 292.5 | 293.1 | 295.0 | 298.0 | 303.0 | 308.5 | 314.0 | 319.0 | 322.0 | 323.9 | 324.5 |
| **36** | 297.0 | 297.6 | 299.6 | 302.7 | 307.8 | 313.5 | 319.1 | 324.2 | 327.3 | 329.3 | 330.0 |
| **37** | 300.8 | 301.5 | 303.5 | 306.7 | 312.0 | 317.8 | 323.6 | 328.9 | 332.1 | 334.1 | 334.8 |
| **38** | 304.1 | 304.8 | 306.8 | 310.1 | 315.6 | 321.5 | 327.5 | 332.9 | 336.2 | 338.3 | 339.0 |
| **39** | 306.7 | 307.4 | 309.5 | 312.9 | 318.5 | 324.6 | 330.8 | 336.4 | 339.7 | 341.9 | 342.6 |
| **40** | 308.7 | 309.4 | 311.6 | 315.1 | 320.8 | 327.1 | 333.4 | 339.1 | 342.6 | 344.8 | 345.5 |
|  |  |  |  |  |  |  |  |  |  |  |  |

Table S4

Abdominal circumference in mm

| **Weeks of gestation** | **Percentiles** | | | | | | | | | | |
| --- | --- | --- | --- | --- | --- | --- | --- | --- | --- | --- | --- |
|  | **P2.3** | **P3** | **P5** | **P10** | **P25** | **P50** | **P75** | **P90** | **P95** | **P97** | **P97.7** |
| **15** | 83.6 | 83.7 | 84.3 | 85.2 | 86.8 | 88.4 | 90.1 | 91.6 | 92.5 | 93.1 | 93.3 |
| **16** | 94.6 | 94.8 | 95.5 | 96.5 | 98.3 | 100.2 | 102.2 | 103.9 | 105.0 | 105.7 | 105.9 |
| **17** | 105.5 | 105.8 | 106.5 | 107.7 | 109.7 | 111.9 | 114.1 | 116.1 | 117.3 | 118.1 | 118.4 |
| **18** | 116.3 | 116.6 | 117.4 | 118.8 | 121.0 | 123.5 | 125.9 | 128.2 | 129.6 | 130.4 | 130.7 |
| **19** | 126.9 | 127.2 | 128.2 | 129.7 | 132.2 | 134.9 | 137.6 | 140.1 | 141.6 | 142.6 | 142.9 |
| **20** | 137.4 | 137.8 | 138.8 | 140.5 | 143.2 | 146.2 | 149.2 | 151.9 | 153.6 | 154.6 | 154.9 |
| **21** | 147.8 | 148.1 | 149.3 | 151.1 | 154.0 | 157.3 | 160.6 | 163.5 | 165.3 | 166.5 | 166.9 |
| **22** | 157.9 | 158.4 | 159.6 | 161.5 | 164.7 | 168.3 | 171.8 | 175.0 | 177.0 | 178.2 | 178.6 |
| **23** | 168.0 | 168.4 | 169.7 | 171.8 | 175.3 | 179.1 | 182.9 | 186.3 | 188.4 | 189.7 | 190.2 |
| **24** | 177.8 | 178.3 | 179.7 | 181.9 | 185.6 | 189.7 | 193.8 | 197.4 | 199.7 | 201.1 | 201.6 |
| **25** | 187.5 | 188.0 | 189.5 | 191.9 | 195.8 | 200.1 | 204.5 | 208.4 | 210.8 | 212.3 | 212.8 |
| **26** | 196.9 | 197.5 | 199.1 | 201.6 | 205.8 | 210.4 | 215.0 | 219.2 | 221.7 | 223.3 | 223.8 |
| **27** | 206.2 | 206.8 | 208.5 | 211.1 | 215.6 | 220.4 | 225.3 | 229.7 | 232.4 | 234.1 | 234.6 |
| **28** | 215.3 | 215.9 | 217.7 | 220.5 | 225.2 | 230.3 | 235.4 | 240.1 | 242.9 | 244.7 | 245.3 |
| **29** | 224.1 | 224.8 | 226.6 | 229.6 | 234.5 | 239.9 | 245.3 | 250.2 | 253.2 | 255.0 | 255.7 |
| **30** | 232.8 | 233.5 | 235.4 | 238.5 | 243.7 | 249.3 | 255.0 | 260.1 | 263.3 | 265.2 | 265.9 |
| **31** | 241.2 | 241.9 | 243.9 | 247.2 | 252.6 | 258.5 | 264.4 | 269.8 | 273.1 | 275.1 | 275.8 |
| **32** | 249.4 | 250.1 | 252.2 | 255.6 | 261.3 | 267.5 | 273.7 | 279.3 | 282.7 | 284.8 | 285.6 |
| **33** | 257.3 | 258.1 | 260.3 | 263.8 | 269.7 | 276.2 | 282.6 | 288.5 | 292.1 | 294.3 | 295.1 |
| **34** | 265.0 | 265.8 | 268.1 | 271.8 | 277.9 | 284.6 | 291.4 | 297.5 | 301.2 | 303.5 | 304.3 |
| **35** | 272.4 | 273.2 | 275.6 | 279.5 | 285.8 | 292.8 | 299.8 | 306.2 | 310.0 | 312.4 | 313.3 |
| **36** | 279.5 | 280.4 | 282.9 | 286.9 | 293.5 | 300.8 | 308.0 | 314.6 | 318.6 | 321.1 | 322.0 |
| **37** | 286.4 | 287.3 | 289.9 | 294.1 | 300.9 | 308.4 | 315.9 | 322.8 | 326.9 | 329.5 | 330.4 |
| **38** | 293.0 | 293.9 | 296.6 | 300.9 | 308.0 | 315.8 | 323.6 | 330.7 | 335.0 | 337.6 | 338.6 |
| **39** | 299.3 | 300.3 | 303.1 | 307.5 | 314.8 | 322.9 | 330.9 | 338.3 | 342.7 | 345.5 | 346.4 |
| **40** | 305.3 | 306.3 | 309.2 | 313.8 | 321.4 | 329.7 | 338.0 | 345.6 | 350.2 | 353.0 | 354.0 |
|  |  |  |  |  |  |  |  |  |  |  |  |

Table S5

Femur length in mm

| **Weeks of gestation** | **Percentiles** | | | | | | | | | | |
| --- | --- | --- | --- | --- | --- | --- | --- | --- | --- | --- | --- |
|  | **P2.3** | **P3** | **P5** | **P10** | **P25** | **P50** | **P75** | **P90** | **P95** | **P97** | **P97.7** |
| **15** | 15.5 | 15.6 | 15.7 | 16.1 | 16.6 | 17.2 | 17.8 | 18.3 | 18.6 | 18.8 | 18.9 |
| **16** | 18.5 | 18.5 | 18.7 | 19.1 | 19.6 | 20.2 | 20.8 | 21.4 | 21.7 | 21.9 | 22.0 |
| **17** | 21.4 | 21.5 | 21.7 | 22.0 | 22.6 | 23.2 | 23.8 | 24.4 | 24.7 | 25.0 | 25.0 |
| **18** | 24.3 | 24.3 | 24.6 | 24.9 | 25.5 | 26.2 | 26.8 | 27.4 | 27.7 | 28.0 | 28.0 |
| **19** | 27.1 | 27.2 | 27.4 | 27.8 | 28.4 | 29.0 | 29.7 | 30.3 | 30.7 | 30.9 | 31.0 |
| **20** | 29.8 | 29.9 | 30.2 | 30.5 | 31.2 | 31.9 | 32.6 | 33.2 | 33.6 | 33.8 | 33.9 |
| **21** | 32.5 | 32.6 | 32.9 | 33.3 | 33.9 | 34.6 | 35.4 | 36.0 | 36.4 | 36.6 | 36.7 |
| **22** | 35.2 | 35.3 | 35.5 | 35.9 | 36.6 | 37.3 | 38.1 | 38.7 | 39.2 | 39.4 | 39.5 |
| **23** | 37.7 | 37.8 | 38.1 | 38.5 | 39.2 | 40.0 | 40.7 | 41.4 | 41.8 | 42.1 | 42.2 |
| **24** | 40.2 | 40.3 | 40.6 | 41.0 | 41.8 | 42.5 | 43.3 | 44.0 | 44.5 | 44.7 | 44.8 |
| **25** | 42.7 | 42.8 | 43.0 | 43.5 | 44.2 | 45.0 | 45.8 | 46.6 | 47.0 | 47.3 | 47.4 |
| **26** | 45.0 | 45.1 | 45.4 | 45.9 | 46.6 | 47.4 | 48.3 | 49.0 | 49.5 | 49.8 | 49.9 |
| **27** | 47.3 | 47.4 | 47.7 | 48.1 | 48.9 | 49.8 | 50.6 | 51.4 | 51.9 | 52.2 | 52.3 |
| **28** | 49.5 | 49.6 | 49.9 | 50.4 | 51.2 | 52.0 | 52.9 | 53.7 | 54.2 | 54.5 | 54.6 |
| **29** | 51.6 | 51.7 | 52.0 | 52.5 | 53.3 | 54.2 | 55.1 | 55.9 | 56.4 | 56.7 | 56.8 |
| **30** | 53.6 | 53.7 | 54.0 | 54.5 | 55.3 | 56.3 | 57.2 | 58.0 | 58.5 | 58.8 | 59.0 |
| **31** | 55.5 | 55.6 | 55.9 | 56.5 | 57.3 | 58.3 | 59.2 | 60.1 | 60.6 | 60.9 | 61.0 |
| **32** | 57.3 | 57.4 | 57.8 | 58.3 | 59.2 | 60.1 | 61.1 | 62.0 | 62.5 | 62.8 | 63.0 |
| **33** | 59.0 | 59.2 | 59.5 | 60.0 | 60.9 | 61.9 | 62.9 | 63.8 | 64.4 | 64.7 | 64.8 |
| **34** | 60.7 | 60.8 | 61.1 | 61.7 | 62.6 | 63.6 | 64.6 | 65.6 | 66.1 | 66.5 | 66.6 |
| **35** | 62.2 | 62.3 | 62.7 | 63.2 | 64.2 | 65.2 | 66.2 | 67.2 | 67.7 | 68.1 | 68.2 |
| **36** | 63.6 | 63.7 | 64.1 | 64.7 | 65.6 | 66.7 | 67.7 | 68.7 | 69.3 | 69.6 | 69.8 |
| **37** | 64.9 | 65.0 | 65.4 | 66.0 | 67.0 | 68.0 | 69.1 | 70.1 | 70.7 | 71.1 | 71.2 |
| **38** | 66.1 | 66.2 | 66.6 | 67.2 | 68.2 | 69.3 | 70.4 | 71.4 | 72.0 | 72.4 | 72.5 |
| **39** | 67.1 | 67.3 | 67.7 | 68.3 | 69.3 | 70.4 | 71.6 | 72.6 | 73.2 | 73.6 | 73.7 |
| **40** | 68.1 | 68.2 | 68.6 | 69.3 | 70.3 | 71.4 | 72.6 | 73.6 | 74.3 | 74.7 | 74.8 |
|  |  |  |  |  |  |  |  |  |  |  |  |
